# Three versions of Perceived Stress Scale: validation in a sample of Chinese cardiac patients who smoke

**DOI:** 10.1186/1471-2458-10-513

**Published:** 2010-08-25

**Authors:** Doris YP Leung, Tai-hing Lam, Sophia SC Chan

**Affiliations:** 1 School of Nursing, The University of Hong Kong, LKS Faculty of Medicine Building, Pokfulam, Hong Kong; 2 Department of Community Medicine, School of Public Health, The University of Hong Kong, LKS Faculty of Medicine Building, Pokfulam, Hong Kong

## Abstract

**Background:**

Smoking causes heart disease, the major cause of death in China and Hong Kong. Stress is one major trigger of smoking and relapse, and understanding stress among smoking cardiac patients can therefore help in designing effective interventions to motivate them to quit. The objective of this study was to examine the psychometric properties of the Perceived Stress Scale (PSS), and to compare the appropriateness of the three versions of the scale (PSS-14, PSS-10, and PSS-4) among Chinese cardiac patients who were also smokers.

**Methods:**

From March 2002 to December 2004, 1860 cardiac patients who smoked were recruited at the cardiac outpatient clinics of ten acute hospitals in Hong Kong, and 1800 questionnaires were analysed. Participants completed a questionnaire including the PSS, nicotine dependence and certain demographic variables. The psychometric properties of the PSS were investigated: construct validity using confirmatory factor analysis, reliability using Cronbach's alpha and concurrent validity by examining the relationship with smoking- and health-related variables.

**Results:**

For all the three versions of the PSS, confirmatory factor analyses corroborated the 2-factor structure of the scale, with the positive and negative factors correlating significantly and negatively to a moderate extent (*r *< -0.5), and high Cronbach's alpha values for the two subscales (alpha > 0.5). All the correlations of the two subscales and the smoking- and health-related variables were statistically significant and in the expected directions although of small magnitudes, except daily cigarette consumption.

**Conclusions:**

The findings confirmed the satisfactory psychometric properties of all three Chinese versions of PSS. We recommend the use of PSS-10 for research which focuses on the two components of perceived stress, as it shows a higher reliability; and the use of PSS-4 if such partition is not essential and space for multiple measures is limited.

## Background

Stress is a commonly reported trigger of smoking and relapse after quitting. Previous studies have shown that stress increases the amount of smoking, reduces the likelihood of successful quitting, and predicts relapse [1,2]. Higher stress is associated with less self-efficacy in quitting and less in not smoking among working adults [3], and less predicted success in quitting prospectively [4].

Heart disease is a leading cause of death in both China and Hong Kong [5,6]. Smoking is a modifiable health risk-factor for cardiac patients [7], but many of them continue to smoke regardless of the gain in health benefits from quitting including a reduction in both morbidity and mortality. There are calls for smoking cessation interventions which target smokers with high perceived stress levels [8]; this is particularly applicable to patients with chronic diseases who usually have high levels of perceived stress.

Cohen and colleagues developed the original 14-item English version of the Perceived Stress Scale (PSS-14) as a global measure of stress by asking respondents to report whether their lives seem to be unpredictable, uncontrollable or overloaded [9]. The PSS is one of the most frequently used tools to measure stress in chronic conditions and situations often not listed on other life-event scales, such as the Daily Hassles Scale [10] and Daily Stress Inventory [11]. The PSS is also available in two shortened versions of 10 items (PSS-10) and 4 items (PSS-4), with the items selected from the pool of 14 in the original version of PSS. The PSS is considered to be a brief scale measuring perceived stress that can be administered in a few minutes [12].

The scale has been translated into several languages, including Spanish, Swedish, Japanese and Chinese [13-16]. A few studies have examined the psychometric properties of the PSS, with five using principal component analysis (PCA) and three confirmatory factor analysis (CFA). The PCA studies provide important yet limited psychometric information on the PSS, as the statistical method has been criticised by methodologists [17]. Two CFA studies were conducted in clinical populations [18,19] and one focused on college students [13], but mixed results on the structure of the PSS were reported. All the three CFA validation studies found a two-factor structure of the PSS, while others reported a one-factor structure, in particular for the 4-item version [12,20]. Moreover, it is unclear whether all the three versions of the PSS have a similar two-factor structure as well as relationships with certain variables.

The PSS has been applied extensively as a health-related outcome in studies examining the effect of smoking cessation interventions [21,22] and the Chinese version of the PSS has also been used in research areas such as mental health and physical activities [23,24]. Although the Chinese version of PSS was found to have high internal consistency and test-retest reliability, its construct and concurrent validity have not yet been established among smokers in Chinese societies, precluding the confident use of the instrument in this large population of varying cultures. The goals of the present study were to assess the construct validity, reliability and concurrent validity of the three versions of the Perceived Stress Scale (PSS), and to compare their appropriateness when used among Hong Kong Chinese cardiac patients who smoke.

## Methods

### Participants

The participants were the 1860 subjects of a randomised control trial investigating the effectiveness of a smoking cessation intervention among cardiac patients who smoked (Trial registration: http://www.isrctn.org Identifier: ISRCTN32840413). Chinese patients attending the cardiac outpatient clinics of 10 acute hospitals in Hong Kong were eligible if they had smoked at least one cigarette daily over the seven days prior to attendance. Ethical approval was obtained from the Ethics Committee of the Faculty of Medicine, University of Hong Kong and from the participating hospitals. Written consent was obtained from all participants. Recruitment was conducted from March 2002 to December 2004. Details of subject recruitment and the results of the study have been reported elsewhere [25]. Data were collected at baseline using a self-administered standardised and structured questionnaire, with a nurse helping to check for completeness. The sample consisted of 1693 men and 167 women, with a mean age of 58.3 (SD = 14.1); the majority (95.8%) had smoked daily over the past 30 days, with an average of daily consumption of 20 cigarettes (SD = 10). About half of the subjects (51.5%) had coronary heart disease, and the other half had other heart problems such as congenital cardiac disease and arrhythmia.

### Measures

The Perceived Stress Scale consists of 14 items (PSS-14) [9], seven positive and seven negative, and was translated into Chinese by the research team (see Additional file 1). Care was taken to ensure that each translated item retained a meaning as close as possible to the original version. The negative element is intended to assess lack of control and negative affective reactions, while the positive element measures the degree of ability to cope with existing stressors. Each item is rated on a five-point scale from 0 = '*never' *to 4 = '*very often'*, covering the preceding month. The 10-item version (PSS-10) consists of six negative and four positive items, while the 4-item version (PSS-4) has two items for each of the two factors.

The Fagerström Test of Nicotine Dependency (FTND) [26], with six questions, was used to measure nicotine dependency. Responses are summed to compute a score ranging from 0 to 10. A higher FTND score indicates a greater degree of dependency. The Chinese version of FTND has been validated [27] and previously used in smoking cessation studies [28]. The Cronbach's alpha coefficient of FTND is 0.76 of the sample in the current study. We also assessed subjects' daily cigarette consumption over the past month, perceived health status over the past three months, confidence in not smoking again, and anxiety and depression at baseline. Each aspect was measured by a single item, with higher scores indicating better health, more confidence, more anxiety and more depression.

### Data analysis

The factor structure of the three PSS versions was examined by confirmatory factor analysis of the 1800 subjects who completed the PSS. A one-factor model with all the items as indicators and a two-factor model with items corresponding to the positive and negative factors were fitted to the covariance matrix of the corresponding PSS items. The reliability of the scale was assessed by Cronbach's alpha, and concurrent validity was evaluated by correlations of the positive and negative PSS factors with FTND scores, daily cigarette consumption over the past month, and perceived health status, anxiety and depression. Furthermore, for each of the three versions of PSS, levels of stress were compared between males and females, using t-tests for the total scores and MANOVA for the scores of the positive and negative subscales simultaneously. Total stress scores were computed by first reversing the scores of the positive items and then summing all the items of the PSS. Sixty subjects left one or more items blank in the complete PSS version, and were excluded from the analysis.

All the CFAs were performed using EQS 6.0 and the maximum likelihood estimation method [29]. Model evaluations were made using a variety of fit indices, including the comparative fit index (CFI) [30], standardized root mean square residual (SRMR) [29], and the root mean square error of approximation (RMSEA) [31]. Values of CFI > 0.9, SRMR < 0.08, and RMSEA < 0.08 are indicative of a good fit with the data [32]. Model chi-square test statistics and associated degrees of freedom and p-values were reported for completeness, although they were not used in model evaluation [33]. Cronbach's alpha, correlation, t-tests and MANOVA were computed using SPSS16.0. All statistical tests were two-tailed, and results were considered significant at *p *< 0.05.

## Results

### Confirmatory factor analysis

Examination of the fit indexes in Table 1 reveals that the 2-factor models fitted well with PSS-10 and PSS-4 but only marginally with PSS-14, while the 1-factor models did not provide acceptable fits with any of the three PSS versions. Table 2 presents the standardised factor loadings of the two-factor models for PSS-14, PSS-10 and PSS-4. For PSS-14, all standardised factor loadings were statistically significant and > 0.4, except those associated with items 11 and 12 of the negative factor. Similar results were observed for PSS-10: all the standardised factor loadings were > 0.4 except item 11 of the negative factor. For PSS-4, all four standardised factor loadings were > 0.4. The two factors were statistically significant and correlated negatively, to a moderate extent, for all the three versions of PSS.

**Table 1:** Results of confirmatory factor analyses of model testing of PSS-14, PSS-10 and PSS-4

	χ^2^	df	p-value	CFI	RMSEA	SRMR
PSS-14						
1-factor model	1341.7	77	< 0.001	0.747	0.096	0.096
2-factor model	557.2	76	< 0.001	0.904	0.059	0.059
PSS-10						
1-factor model	886.0	35	< 0.001	0.762	0.116	0.091
2-factor model	198.9	34	< 0.001	0.954	0.052	0.048
PSS-4						
1-factor model	80.9	2	< 0.001	0.900	0.147	0.068
2-factor model	7.0	1	< 0.001	0.992	0.014	0.057

CFI = Comparative Fit Index; RMSEA = root mean square error of approximation; SRMR = Standardised root mean square residuals.

**Table 2:** Standardised factor loadings of the 2-factor models fitted to PSS-14, PSS-10 and PSS-4.

		PSS-14		PSS-10		PSS-4	
		
Item		Negative	Positive	Negative	Positive	Negative	Positive
1	upset by something happening unexpectedly?	.76	0	.77	0	-	-
2	unable to control the important things in your life?	.81	0	.82	0	.74	0
3	nervous and stressed?	.79	0	.78	0	-	-
8	could not cope with all the things that you had to do?	.44	0	.44	0	-	-
11	angered because of things that happened that were outside your control?	.27	0	.26	0	-	-
12	thinking about things that you have to accomplish?	.34	0	-	-	-	-
14	difficulties were pilling up so high that you could not overcome them?	.44	0	.44	0	.46	0

4	dealt successfully with day-to-day problems and annoyances?	0	.64	-	-	-	-
5	effectively coping with important changes that were occurring in your life?	0	.66	-	-	-	-
6	confident about your ability to handle your personal problems?	0	.79	0	.77	0	.73
7	things were going your way?	0	.72	0	.77	0	.86
9	dealt successfully with irritating life hassles?	0	.66	0	.63	-	-
10	you were on top of things?	0	.74	0	.78	-	-
13	able to control the way you spend your time?	0	.56	-	-	-	-

	Factor correlation	-.54		-.57		-.58	

	Cronbach alpha	.77	.86	.76	.83	.51	.77
		.85		.83		.67	

### Reliability analysis

The coefficient alpha values for the positive and negative subscales were 0.86 and 0.77 for PSS-14, 0.83 and 0.76 for PSS-10, and 0.77 and 0.51 for PSS-4, respectively. All exceeded Kline's criterion of 0.7 for internal consistency [34], except in the case of the negative subscale of PSS-4. The Cronbach's alpha values of the full scales ranged from 0.67 for the PSS-4 to 0.85 for PSS-14. Scores on the positive and negative subscales were computed by averaging the corresponding items for PSS-14, PSS-10, and PSS-4 in each case; higher scores on negative and positive subscales indicate higher levels of perceived distress and coping ability, separately. Overall scores for the three versions of PSS then were computed by adding the negative and the reverse of the positive subscale scores.

### Concurrent validity

As shown in Table 3, the positive subscale correlated negatively with FTND scores and levels of anxiety and depression, and positively with daily cigarette consumption and perceived health; while the negative subscale showed an opposite direction of the correlations with these variables. The pattern of correlations for the three overall PSS scores was similar to that of the negative subscales. For all three versions of the PSS, all correlations were statistically significant and in the expected direction, with one exception: daily cigarette consumption correlated significantly and positively with the positive factor, but negatively with the negative factor, although the correlation was non-significant. In general, compared with the positive factor, the correlations of the negative factor were larger with health-related variables, similar with FTND scores and smaller with confidence in not smoking again and daily cigarette consumption.

**Table 3:** Correlations of subscale scores on PSS-14, PSS-10 and PSS-4 and smoking and health related variables

	PSS-14			PSS-10			PSS-4		
	
	-ve Factor	+ve Factor	Overall	-ve Factor	+ve Factor	Overall	-ve Factor	+ve Factor	Overall
**Smoking-related variables**									
Daily cigarette consumption in the past month	-.04	.09**	.01	-.03	.09**	.03	.00	.08**	.05*
FTND score^1^	.10**	-.09**	.11**	.10**	-.10**	.11**	.07*	-.11**	.10**
Confidence in not smoking again^2^	-.14**	.20**	-.19**	-.13**	.23**	-.19**	-.91**	.21**	-.18**
**Health-related variables**									
Perceived health status in the past 3 months^3^	-.15**	.11**	-.16**	-.16**	.12**	-.17**	-.17**	.13**	-.19**
Anxiety today^4^	.21**	-.10**	.18**	.20**	-.12**	.19**	.19**	-.13**	.19**
Depression today^5^	.22**	-.16**	.22**	.23**	-.19**	.24**	.22**	-.19**	.24**

^1 ^FTND scores range from 0 to 10, with higher values indicating higher levels of nicotine dependency.

^2 ^Confidence in not smoking again was measured with a scale from 0-100, with higher values indicating higher levels of confidence

^3 ^Perceived health status was measured on a 4-point Likert scale, with higher values indicating better perceived health.

^4 ^Anxiety was measured on a 3-point Likert scale, with higher values indicating higher levels of anxiety.

^5 ^Depression was measured on a 3-point Likert scale, with higher values indicating higher levels of depression.

* p-value < 0.05 ** p-value < 0.01

### Comparison of stress level by sex

Table 4 shows the descriptive, t-test and MANOVA results of the sex effect on perceived stress levels, as measured by PSS-14, PSS-10 and PSS-4. Women reported a significantly higher total mean score on perceived stress than men. In addition, women scored both significantly higher in the negative subscale and lower in the positive subscale than men, irrespective of the PSS version.

**Table 4:** Means of total and subscale scores on PSS-14, PSS-10 and PSS-4 by sex

	Men (n = 1634)		Women (n = 166)		
		
Variable	Mean	SD	Mean	SD	p-value^1^
PSS-14					
Total	22.3	5.7	23.6	6.0	0.006
Positive subscale	15.6	3.4	14.9	3.3	0.021
Negative subscale	9.9	3.3	10.3	3.7	
PSS-10					
Total	15.2	4.4	16.3	4.8	0.003
Positive subscale	8.9	2.1	8.5	2.0	0.010
Negative subscale	8.1	3.0	8.7	3.5	
PSS-4					
Total	6.0	2.0	6.5	2.1	0.007
Positive subscale	4.4	1.2	4.3	1.2	0.014
Negative subscale	2.5	1.2	2.7	1.3	

^1 ^T-tests used for comparing mean differences in the total score, and MANOVA for comparing mean differences in the positive and negative subscale scores simultaneously.

## Discussion

This study has explored and extended the factor structure of the Chinese version of the Perceived Stress Scale and its shorter variants (PSS-10 and PSS-4) from clinic populations in the West to a Chinese cardiac population with a smoking habit. The results show that construct validity, internal consistency and concurrent validity of the Chinese versions of PSS-14, PSS-10 and PSS-4 and their corresponding subscales were generally supported in a community-based sample of Chinese smoking adults with cardiac disease.

Our study provides evidence to support the manifestation of the PSS as two-dimensional, and that the two-factor models give better approximations of the data of PSS-14, PSS-10 and PSS-4 with items loaded on their designated factor. The results obtained are consistent with previous CFA studies using PSS-14 and PSS-12 [13,18,19], but contrary to those using PSS-4, which was hypothesised as one latent factor [12,20]. The factor loading of item 11 of the negative factor was found in the present study to be far below 0.4 in both PSS-14 (0.27) and PSS-10 (0.26). The low factor loadings of this item compared with the rest of the negative factor could be due to the potential interpretation of 'anger' as expressing the perceived distress externally by the subjects. Alternatively, it could simply have been produced by the translation process but we could not find any study utilizing the Chinese PSS reporting the associated factor loadings for comparison and hence more studies are needed to verify this possibility. Indeed, a low factor loading of 0.28 for item 7 has also been reported among a sample of ambulatory care patients with asthma in the US [19]. Future studies are warranted to address equivalence in items of the PSS across sub-populations such as sex, ethnicity and education.

Cronbach's alpha values demonstrate that each of the two subscales of PSS-14 and PSS-10 are internally reliable and form a cohort set of the construct. Similar to previous studies conducted in the Asian region, including Japan, Singapore and Taiwan [24,35,36], high internal consistency reliability of the three overall scales of the PSS was also observed in the current study. Although the alpha value of this subscale of PSS-4 was below the cut-off point of 0.7, it has been argued that a reliability coefficient as low as 0.5 should not seriously attenuate validity [37] and the alpha coefficient increases with the instrument's length [38]; this subscale is therefore still considered reliable.

The subscales of the PSS correlate with smoking- and health-related variables in the anticipated directions except daily cigarette consumption, providing some preliminary evidence on the concurrent validity of the PSS scale in the three versions. Our results on the low correlations of the PSS with smoking-related variables were consistent with previous studies that low and insignificant correlations of the PSS scores with other smoking-related variables were also reported in Cohen and Lichtenstein's study among smokers [1]. In the large community-based study in the US, high stress was significantly associated with reports of increased tobacco consumption and less confidence in not smoking again but the magnitude of the association was also small after controlled for demographic variables [3]. Results from our study and the two previous studies show that PSS is associated with smoking-related variables but it might not act as a proxy for them.

The opposite correlations of the positive and negative factors of PSS with these variables provide further evidence of the manifestation of two components in PSS. The greater correlations with 'perceived distress' than with 'perceived coping' support the assertion that individuals react to a stressor or threatening event with a primary appraisal, and judge their ability to cope with the stressor or event with a secondary appraisal in the stress and coping model [39]. Further research will need to test the differential predictive validity of the two PSS subscales.

Consistent with earlier findings [40,41], we have also found that women reported a significant higher total score for perceived stress. In addition, we have found women had significantly higher scores in the negative and lower scores in the positive subscales than men, regardless of the PSS version. This result is also consistent with previous findings, that women smoke more for tension reduction and relaxation [42,43].

Our results show that the psychometric properties of all three versions are satisfactory and similar. However, it is important to consider which version of the PSS should be used in particular applications. As in a previous study [40], the PSS-10 not only provides an adequate measure of perceived stress and similar correlations with smoking- and health-related measures as the complete version, but has also shown a higher reliability among our Chinese patients. In addition, the PSS-4 also has satisfactory construct validity, but a somewhat lower reliability in the negative subscale. From a practical point of view, because a shorter questionnaire would be more appropriate for studies with multiple measures, we recommend the use of PSS-10 in future research to focus on the two components of perceived stress; and the use of PSS-4 if such separation is not essential and space for multiple measures is limited [44].

This study had several limitations. First, the present validation study of the PPS was part of a randomised controlled trial carried out among a group of cardiac outpatients who smoke. Given the large sample size, our results can be generalised to smoking cardiac patients, but not to those who do not smoke. Moreover, the large proportion of male participants in the study may limit its generalisability to female smokers. However, the potential of the current findings to be generalised is enormous, given that smoking in Hong Kong and elsewhere in Asia is strongly sex-linked with much higher smoking prevalence in men (men >40%, women <10%) [45] and that cardio-vascular disease is the number one killer in China [6]. Nevertheless, further research using population-based study and on different subgroups among the Chinese and other Asian populations is warranted. Second, the use of single-item measures of smoking and health-related variables in the study (except FTND) should include a substantial amount of measurement error in each of these variables. The imperfect measures of these variables however would lead to underestimation of the correlations with other variables, which could explain the low observed correlations with the PSS scores [46]. Third, we were unable to assess test-retest reliability of the PSS in the current study as it was a secondary data analysis of an RCT and such information were not collected at follow-up surveys. Further studies on the psychometric properties of the Chinese versions of the PSS should be carefully designed to include both precise measures of potential related variables to assess concurrent validity and a test-retest component to examine stability of the instrument.

## Conclusion

The study corroborates the observations of others in demonstrating that the Chinese-translated PSS has adequate construct validity, reliability and concurrent validity, and hence is acceptable in a clinical smoking population with cardiac disease. Researchers may choose the shorter versions of PSS (PSS-10 and PSS-4) depending on the practical needs of their work.

## Competing interests

The authors declare that they have no competing interests.

## Authors' contributions

DL performed the statistical analysis, interpreted the results and drafted the manuscript. THL participated in the design of the study, and helped to draft and revise the manuscript. SC participated in both design and coordination, and helped to draft and revise the manuscript. All authors reviewed and approved the manuscript.

## Pre-publication history

The pre-publication history for this paper can be accessed here:

http://www.biomedcentral.com/1471-2458/10/513/prepub

## Supplementary Material

Additional file 1 **The Chinese version of the Perceived Stress Scale**. Chinese translation of the 14 items in the Perceived Stress Scale. Click here for file
