# Supplementary material for: Three versions of Perceived Stress Scale: validation in a sample of Chinese cardiac patients who smoke
Source: BMC Public Health. 2010 Aug 25;10:513. doi: 10.1186/1471-2458-10-513 (PMC2939644; doi:10.1186/1471-2458-10-513)
Supplement: Additional file 1 — The Chinese version of the Perceived Stress Scale. Chinese translation of the 14 items in the Perceived Stress Scale. [file 1471-2458-10-513-S1.DOC]

**The Chinese version of the Perceived Stress Scale**

以下問題是問關於你上個月的感受和想法。 每一條題目都是問你“幾經常”有所描述的感受和想法。 雖然有些題目意思看來十分相近，其實它們是不同的。 你應視它們為獨立的題目作答。 最適合的方法是盡快回答每條問題。 不用準確計算次數的多少，只要作出合理的估計。

| 絕對不會 | 大概  不會 | 有時會 | 經常會 | 十分  經常會 |
| --- | --- | --- | --- | --- |

# 1)上個月你有幾經常對某些突然發生的事情感到不安？ 0 1 2 3 4

# 2)上個月你有幾經常感覺到總是沒法控制生活上重要的事？ 0 1 2 3 4

# 3)上個月你有幾經常感覺到焦慮和壓力？ 0 1 2 3 4

# 4)上個月你有幾經常成功地處理生活上令人煩燥的事？ 0 1 2 3 4

# 5)上個月你有幾經常感覺到有效地處理生活上的重大轉變？ 0 1 2 3 4

# 6)上個月在處理個人問題之能力方面，你有幾經常感到充滿信心？ 0 1 2 3 4

# 7)上個月你有幾經常感覺到事事順利？？ 0 1 2 3 4

# 8)上個月你有幾經常發現你是沒法處理各樣應要做的事？ 0 1 2 3 4

# 9)上個月你有幾經常能控制生活上之煩燥？ 0 1 2 3 4

# 10)上個月你有幾經常感到事事駕輕就熟？ 0 1 2 3 4

# 11)上個月你有幾經常對某些屬於你控制範圍以外的事而發怒？ 0 1 2 3 4

# 12)上個月你有幾經常在思想一些務要達到的事？ 0 1 2 3 4

# 13)上個月你有幾經常能控制你對時間的分配？ 0 1 2 3 4

# 14)上個月你有幾經常感覺到有很多困難而未能克服？ 0 1 2 3 4
